# A Bilevel Programming Approach for Optimizing Multi-Satellite Collaborative Mission Planning

**DOI:** 10.3390/s24196242

**Published:** 2024-09-26

**Authors:** Yi Wang, Desheng Liu

**Affiliations:** National Key Laboratory of Space Target Awareness, Space Engineering University, Beijing 101416, China; ttsc_wy@hgd.edu.cn

**Keywords:** multi-satellite collaboration, mission planning, bilevel programming, nested genetic algorithm

## Abstract

With the burgeoning of remote sensing and space technology, multi-satellite collaborative mission planning, which is the key to achieving efficient Earth observation, has become increasingly intricate due to the expanding complexity and volume of observation missions. Addressing the multi-satellite collaborative mission planning problem, which is characterized by its two-stage decision-making process involving mission assignment and resource scheduling, this study investigates a comprehensive joint decision making that encompasses both mission assignment and resource scheduling and comprehensively optimizes the mission completion rate, the mission profit rate, and the satellite resource utilization rate. Considering the interaction of these decisions, we formulate the problem as a bilevel programming model from a game-theoretic perspective and propose a nested bilevel improved genetic algorithm (NBIGA) for its solution. Simulation experiments substantiate the applicability of the bilevel programming model in joint decision making for the stages of mission assignment and resource scheduling in multi-satellite collaborative mission planning, as well as the robustness of the NBIGA. A comparative analysis with the nested bilevel genetic algorithm (NBGA) confirms that the algorithm proposed in this study can achieve superior optimization outcomes and higher solving efficiency.

## 1. Introduction

With the vigorous development of space science and technology worldwide, increasing numbers of Earth observation satellites are in orbit, performing various remote sensing missions, such as target detection and identification, regional target search, and moving target tracking. Due to factors such as the observation range, observation time, and observation resolution, a single satellite often faces difficulty in independently completing an increasing number of observation missions with increasingly complex requirements. However, multiple satellites can more effectively meet the requirements of complex observation missions through collaborative observation. Therefore, optimizing resource allocation to maximize observation benefits and achieving accurate and efficient multi-satellite collaborative mission scheduling is a vital focus in the field of current remote sensing research.

Multi-satellite collaborative mission scheduling is a typical large-scale and complex optimization problem that needs to consider multi-objective requirements, including mission completion, observation benefit, and timeliness of observations, and subject to constraints such as satellite resource capabilities, mission target demands, and related temporal relationships. This problem exhibits features of the diversity of elements, the coupling of relationships, and Non-deterministic Polynomial hard (NP-hard) characteristics, the optimization of which involves a sophisticated process that requires the integration and coordination of various objectives and constraints to achieve an efficient mission planning strategy. The optimization of multi-satellite collaborative mission planning requires two main issues to be addressed: the mission assignment problem, i.e., facing the heavy demand for observation missions, the question of how to assign missions to satellites, achieving high mission-related benefits and, meanwhile, more balanced satellite resource loads; the resource scheduling problem, i.e., the question of how to schedule satellites to determine the specific execution time of missions, ensuring mission completion and benefits and, meanwhile, completing the observation as early as possible to respond more quickly to the user’s needs [1]. Furthermore, the mission assignment problem is that of finding the best combination of mission targets and satellite resources, while the resource scheduling problem is that of finding the best combination of satellite resources and time windows, both of which are combinations of discrete variables. Therefore, multi-satellite collaborative mission scheduling is essentially a kind of complex combinatorial optimization problem [2]. The evidence shows that mission assignment and resource scheduling are inseparable, mutually restrictive, and mutually complementary. The decision of mission assignment is an indispensable input for determining the specific execution time of the mission in the stage of resource scheduling. At the same time, whether the mission can be actually executed by satellites may, in turn, change the decision of mission assignment. To maximize the benefits of observation, the interconnection between the two issues should be considered for joint decision making. However, the complexity of multi-satellite collaborative mission planning makes the joint decision making of mission assignment and resource scheduling, which involve interactive relationships, even more difficult. Previous studies [3,4,5,6,7] adopted a hierarchical decoupling methodology to address the two stages of mission assignment and resource scheduling for this problem. Although commendable planning outcomes were obtained in all of these studies, the resource scheduling optimization was performed after the mission assignment scheme was determined, and there was no synchronous and iterative interaction of information during the whole process, which made it difficult to ensure the maximization of global observational efficiency.

The use of game theory, which studies cooperation and competition among rational decision makers, has become widespread in recent years to solve problems such as mission assignment [8,9,10] and scheduling optimization [11,12,13]. Indeed, the decision-making process of mission assignment and resource scheduling in multi-satellite collaborative mission planning is a game. During the mission assignment stage, a certain goal determines the mission assignment plan, where there may be multiple different execution opportunities for the same mission. When it comes to the resource scheduling stage, considering the satellites’ capabilities and related constraints, decisions are made with certain objectives based on the mission assignment scheme. The decision-making process in the mission assignment stage is not only restricted by its own conditions but also affected by the results of the subsequent resource scheduling stage. Reciprocally, the decisions in the resource scheduling stage are contingent upon the mission assignment determined in the prior stage. This interdependence creates a Stackelberg game-theoretic dynamic balance. This Stackelberg game with a leader–follower relationship is also known as a bilevel programming problem, where the upper level is related to the leader, and the lower level is related to the follower [14]. In the field of satellite mission planning, there are a limited number of studies on the application of bilevel programming to multi-satellite collaborative mission planning, and they rarely consider the connection and interaction between the two stages of mission assignment and resource scheduling. Therefore, this study attempts to optimize the joint decision making in the two stages in multi-satellite collaborative mission planning using a bilevel programming method [15]. In the constructed bilevel programming model, the upper-level model reveals the decision-making process of the mission assignment stage, while the lower-level model describes the decision-making process of the resource scheduling stage. Because of the dual complexity of multi-satellite collaborative mission planning and the bilevel programming model, it is difficult to obtain analytical solutions through mathematical programming methods. The nested genetic algorithm (NGA) has been widely used to solve bilevel programming models [16]. Therefore, we further propose a nested bilevel improved genetic algorithm (NBIGA) to solve the bilevel programming model for multi-satellite collaborative mission planning in this study. Finally, simulation experiments are used to prove the applicability of the bilevel programming model and the robustness of the NBIGA. The main contributions of this study are summarized as follows.

1. Two-stage joint decision making for mission assignment and resource scheduling in multi-satellite collaborative mission planning is analyzed as a Stackelberg game, and a bilevel programming model is established with the upper level as the leader that decides the mission assignment scheme, and the lower level as the follower that decides the resource scheduling scheme.
2. A nested bilevel improved genetic algorithm is designed to solve the bilevel programming model for multi-satellite collaborative mission scheduling.
3. Extensive simulation experiments were performed, and the potential and applicability of the bilevel programming model and the NBIGA for solving multi-satellite collaborative mission planning were verified through a comparison.

The rest of this article is organized as follows. In Section 2, a bilevel programming model framework for multi-satellite collaborative mission planning is proposed, and then the modeling process of the bilevel programming model is described in detail. Finally, the NBIGA proposed in this study is introduced. Section 3 analyzes the adaptability of the model and the robustness of the algorithm through simulation experiments with 10 satellites and 100 missions. The experiments and comparative analysis of algorithms with different mission scales are discussed in Section 4. The research is summarized in Section 5.

## 2. Materials and Methods

### 2.1. Bilevel Programming Solution Framework

**Figure 1:**
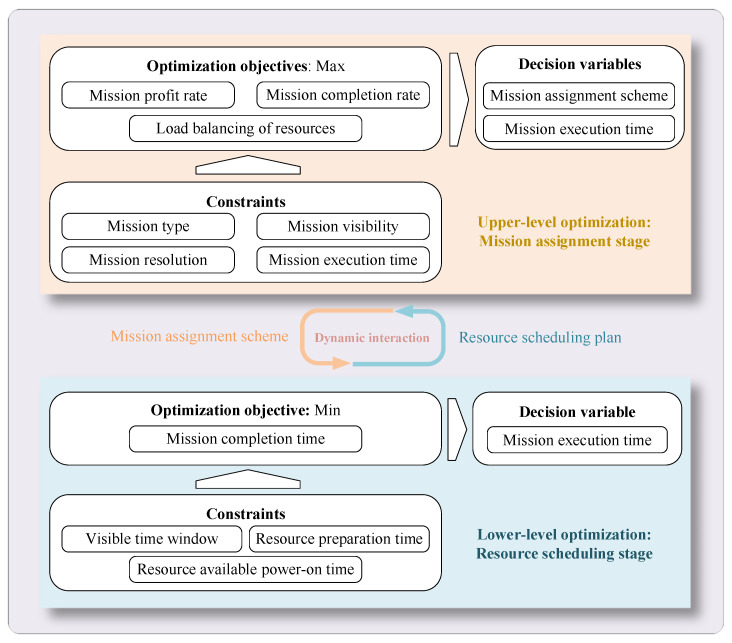
Framework of the proposed bilevel programming model for multi-satellite collaborative mission planning.

In the multi-satellite collaborative mission planning problem, the mission information and satellite data are input in the mission assignment stage, and the optimal mission assignment scheme is the output. In the resource scheduling stage, the input is the mission assignment scheme, and the output is the optimal resource scheduling scheme. The optimizations of the two stages continuously feed back and iterate until an equilibrium is reached. This problem is also a hierarchical decision-making problem where the upper-level multi-mission collaborative assignment and the lower-level multi-satellite collaborative scheduling are interrelated and closely related. The bilevel planning model framework for multi-satellite collaborative mission scheduling is shown in Figure 1.

The upper level is responsible for the decision making in the mission assignment stage, and it mainly aims to maximize the mission completion degree and the benefit rate, as well as to decide the mission assignment scheme under the constraints related to the mission types, resolutions, execution times, and visibility. The lower level is responsible for the decision making in the resource scheduling stage, and it takes minimizing the mission completion time as the optimization goal. Under resource-related constraints, such as the visible time windows, resource preparation time, and resource available power-on time, the resource scheduling scheme is formulated, and meanwhile, the specific execution time of each mission is determined. The framework clearly describes the connection and interaction between the two stages of mission assignment and resource scheduling. The upper level provides the mission assignment plan to the lower level, and the lower level schedules satellites for target observation under the guidance of the plan and feeds the decision results back to the upper level. Then, the upper level adjusts and optimizes the mission assignment plan according to the results.

### 2.2. Bilevel Programming Model Construction

Based on the analysis of the multi-satellite collaborative mission planning problem and the proposed bilevel programming model framework, a bilevel programming model for multi-satellite collaborative mission planning is constructed. The bilevel programming model aims to optimize the allocation of satellite resources and improve the utilization rate of satellite resources under the premise of maximizing the satisfaction of user needs.

#### 2.2.1. Model Assumptions and Simplifications

To avoid the influence of weakly related factors and ensure the wide applicability of this study, the following reasonable assumptions are considered to establish the bilevel programming model.

1. It is assumed that all missions are preprocessed point targets with single payload requirements and schedulable meta-missions with a certain duration.
2. It is assumed that the satellites have autonomous mission planning capabilities and inter-satellite communication capabilities, without considering the impact related to data transmission activities.
3. It is assumed that each satellite carries only one payload, whose fixed storage capacity limit and power limit can be measured with the maximum longest power-on time of the satellite, and one satellite can only execute one mission at a time.
4. The impact of weather and other environmental factors is ignored.

#### 2.2.2. Notation

For ease of subsequent reference, the parameters included in this model and their descriptions are illustrated in Table 1.

#### 2.2.3. Upper-Level Optimization: Mission Assignment

This section describes the construction of the upper-level optimization model, which is a key component in the overall bilevel programming framework. The upper-level optimization is mainly responsible for making global decisions to achieve the overall optimization of multi-satellite collaborative mission scheduling. These decisions will directly affect the input and parameters of the lower-level optimization for resource scheduling and, thus, have a decisive impact on the efficiency of the whole system.

- Optimization objective function

The upper level, as the leader in the whole bilevel programming model, has optimization objectives that not only include the global optimization goals but also regulate the lower-level optimization from a global perspective. The upper-level optimization needs not only to consider the benefits of mission assignment but also to take the load balancing of satellites and the collaborative work among multiple satellites into account. To ensure the efficient completion of missions and the optimal utilization of resources, the optimization objective function is as follows:

(1) $$\begin{array}{l} F_{UL}=\max[a\times \frac{\sum_{i=1}^{m}\sum_{j=1}^{n}y_{ij}\times p_{i}}{\sum_{i=1}^{m}p_{i}}+b\times \frac{\sum_{i=1}^{m}\sum_{j=1}^{n}y_{ij}}{m}+c\times Lb]\\ Lb=1-\sqrt{\frac{1}{n-1}\sum_{j=1}^{n}(\frac{\sum_{i=1}^{m}x_{ij}\times c_{d,i}}{c_{p,j}}-\bar{L}{)}^{2}}\\ \bar{L}=(\sum_{j=1}^{n}\frac{\sum_{i=1}^{m}x_{ij}\times c_{d,i}}{c_{p,j}})/n \end{array}$$

This optimization objective consists of three parts. The first part is the mission profit rate, which represents the rate of the sum of the profits from completed missions to the sum of profits from all missions, indicating the efficiency of mission execution in terms of profit. And the value of this rate fluctuates between 0 and 1. The second part describes the completion of missions, which is the rate of the number of missions completed to the total number of missions, signifying the proportion of the mission objectives that have been successfully fulfilled. The value also ranges between 0 and 1, indicating the completion percentage of the assigned missions. Moreover, the third part represents the load balancing of satellites, ensuring that no single satellite is overburdened, which is crucial for maintaining operational efficiency and longevity of the satellite system. And the load balancing factor also ranges between 0 and 1, where 1 represents a perfectly even distribution of missions across all satellites. The weight coefficients are *a*, *b*, and *c*, respectively, and their sum is equal to 1.

- Constraints

The constraints of the upper-level model are mainly related to the missions, and they include the following.

1. Mission type constraints: The imaging type required for each mission must match the payload type of the satellite executing that mission.
  (2) $$\begin{array}{c} \forall i\in \{1,2,\ldots ,m\},j\in \{1,2,\ldots ,n\},x_{ij}=1\\ c_{q,i}=c_{l,j} \end{array}$$
2. Mission resolution constraints: The observation resolution of each mission should be no less than the minimum resolution of the payload of the satellite executing the mission.
  (3) $$\begin{array}{c} \forall i\in \{1,2,\ldots ,m\},j\in \{1,2,\ldots ,n\},x_{ij}=1\\ c_{r,i}\geq c_{s,j} \end{array}$$
3. Mission visibility constraints: The mission target and the satellite must be visible to each other, i.e., the mission can only be assigned to a satellite that has a visible time window for it.
  (4) $$\begin{array}{c} \forall i\in \{1,2,\ldots ,m\},j\in \{1,2,\ldots ,n\},h\in \{1,2,\ldots ,k\},x_{ij}=1\\ v_{ijh}⊄\emptyset \end{array}$$
4. Mission execution constraints: Each mission can be observed and executed once by one satellite in one of its visible time windows at most.
  (5) $$\begin{array}{c} \forall i\in \{1,2,\ldots ,m\},j\in \{1,2,\ldots ,n\}\\ \sum_{i=1}^{m}\sum_{j=1}^{n}x_{ij}\times y_{ij}\leq 1 \end{array}$$

#### 2.2.4. Lower-Level Optimization: Resource Allocation

As the follower in the bilevel programming model, the lower-level optimization is responsible for resource scheduling to decide the specific execution time of missions under the premise of satisfying the decision of the upper level.

- Optimization objective function

Guided by the global decision of the upper-level model, the optimization objective of the lower-level model is to ensure, as far as possible, that each mission can be completed within the specified time and resource constraints while taking the collaborative effects among multiple satellites into account. To improve the timeliness of mission scheduling, better meet users’ needs, and improve the utilization rate of satellite resources, the optimization goal of the lower level is to minimize the mission completion time. To facilitate the solution, it is converted into the following maximum function:

(6) $$F_{LL}=\max(1-\frac{1}{m}\sum_{i=1}^{m}\frac{o_{i}+c_{d,i}}{period})$$

where *period* is the planning time. Minimization of the mission completion time means that in the process of multi-satellite collaborative mission planning, the end time of the last mission target observation completed is as soon as possible. This optimization objective can promote collaborative work among satellites, make more efficient use of resources, respond to users’ needs more quickly, and improve the overall performance of the multi-satellite system. The lower-level optimization objective complements the global optimization objectives of the upper level, ensuring consistency and coordination from the strategy to the execution level.

- Constraints

The constraints of the lower-level model are mainly related to satellite resources, and they include the following:

1. Visible time window constraints: The duration of the visible time window for the satellite performing the observation mission should meet the minimum observation time requirement of the mission.
  (7) $$\begin{array}{c} \forall i\in \{1,2,\ldots ,m\},j\in \{1,2,\ldots ,n\},h\in \{1,2,\ldots ,k\},y_{ij}=1\\ v_{s,ijh}\leq o_{i},o_{i}+c_{d,i}\leq v_{e,ijh} \end{array}$$
2. Resource preparation time constraints: After the completion of a mission, the satellite needs to adjust its attitude to continue the next mission. When a satellite needs to execute missions continuously in sequence, the time interval between the two missions should not be less than the satellite’s attitude adjustment time.
  (8) $$\begin{array}{c} \forall i\in \{1,2,\ldots ,m\},j\in \{1,2,\ldots ,n\},y_{ij}=1\\ o_{i,next}-o_{i}-c_{d,i}\geq c_{a,j} \end{array}$$
3. Resource maximum power-on time constraints: The cumulative time occupied by all activities of a satellite within the mission planning period shall not exceed the maximum power-on time of the satellite.
  (9) $$\begin{array}{c} \forall i\in \{1,2,\ldots ,m\},j\in \{1,2,\ldots ,n\},y_{ij}=1\\ y_{ij}\times (c_{d,i}+c_{a,j})\leq c_{p,j} \end{array}$$

#### 2.2.5. The Leader–Follower Joint Optimization Model

In this study, the two-stage decision process of mission assignment and resource scheduling is modeled as a bilevel programming model, with the specific mission execution time as the associated variable between the upper and lower levels. The upper-level optimization serves as the leader for mission assignment, while the lower-level optimization serves as the follower for resource scheduling. To sum up, the leader–follower optimization of this decision problem can be formulated as the following bilevel programming model:

(10) $$\begin{array}{l} \underset{r,o}{\max}[a\times \frac{\sum_{i=1}^{m}\sum_{j=1}^{n}y_{ij}\times p_{i}}{\sum_{i=1}^{m}p_{i}}+b\times \frac{\sum_{i=1}^{m}\sum_{j=1}^{n}y_{ij}}{m}+\\ \;\;\;c\times [1-\sqrt{\frac{1}{n-1}\sum_{j=1}^{n}(\frac{\sum_{i=1}^{m}x_{ij}\times c_{d,i}}{c_{p,j}}-(\sum_{j=1}^{n}\frac{\sum_{i=1}^{m}x_{ij}\times c_{d,i}}{c_{p,j}})/n{)}^{2}}]]\\ s.t.\left\{\begin{array}{l} \begin{array}{ll} c_{q,i}=c_{l,j}, & \forall i\in \{1,2,\ldots ,m\},j\in \{1,2,\ldots ,n\},x_{ij}=1 \end{array}\\ \begin{array}{ll} c_{r,i}\geq c_{s,j}, & \forall i\in \{1,2,\ldots ,m\},j\in \{1,2,\ldots ,n\},x_{ij}=1 \end{array}\\ \begin{array}{ll} v_{ijh}⊄\emptyset , & \forall i\in \{1,2,\ldots ,m\},j\in \{1,2,\ldots ,n\},h\in \{1,2,\ldots ,k\},x_{ij}=1 \end{array}\\ \begin{array}{ll} \sum_{i=1}^{m}\sum_{j=1}^{n}x_{ij}\times y_{ij}\leq 1, & \forall i\in \{1,2,\ldots ,m\},j\in \{1,2,\ldots ,n\} \end{array} \end{array}\right.\\ where\;for\;the\;given\;r,\;the\;variable\;o\;solves:\\ \left\{\begin{array}{l} \underset{o}{\max}(1-\frac{1}{m}\sum_{i=1}^{m}\frac{o_{i}+c_{d,i}}{period})\\ s.t.\left\{\begin{array}{l} \begin{array}{ll} v_{s,ijh}\leq o_{i}, & \forall i\in \{1,2,\ldots ,m\},j\in \{1,2,\ldots ,n\},h\in \{1,2,\ldots ,k\},y_{ij}=1 \end{array}\\ \begin{array}{ll} o_{i}+c_{d,i}\leq v_{e,ijh}, & \forall i\in \{1,2,\ldots ,m\},j\in \{1,2,\ldots ,n\},h\in \{1,2,\ldots ,k\},y_{ij}=1 \end{array}\\ \begin{array}{ll} o_{i,next}-o_{i}-c_{d,i}\geq c_{a,j}, & \forall i\in \{1,2,\ldots ,m\},j\in \{1,2,\ldots ,n\},y_{ij}=1 \end{array}\\ \begin{array}{ll} y_{ij}\times (c_{d,i}+c_{a,j})\leq c_{p,j}, & \forall i\in \{1,2,\ldots ,m\},j\in \{1,2,\ldots ,n\},y_{ij}=1 \end{array} \end{array}\right. \end{array}\right. \end{array}$$

In this bilevel programming model, the decision variables ***r*** and ***o*** both represent specific practical significance, the former represents the mission assignment scheme, the latter represents the specific mission execution time. Therefore, when measuring the quality of decision, the two 0–1 numerical variables *x_ij_* and *y_ij_* are introduced to correspond numerically to the mission assignment reflected by the decision variable ***r*** and the resource scheduling reflected by the decision variable ***o***, respectively. the decision sequence is as follows. First, the upper-level optimization decides ***r*** and reflects the mission assignment through the 0–1 numerical variable *x_ij_*. Then, the lower-level optimization decides the specific mission execution time ***o*** based on the mission assignment plan, which directly determines the mission completion *y_ij_*. Subsequently, the upper level further decides ***r*** and ***o*** from the global perspective according to the feedback from the lower level for mission assignment. Finally, through repeated iterations, the optimal multi-satellite collaborative mission scheduling scheme is output.

### 2.3. A Nested Bilevel Improved Genetic Algorithm

**Figure 2:**
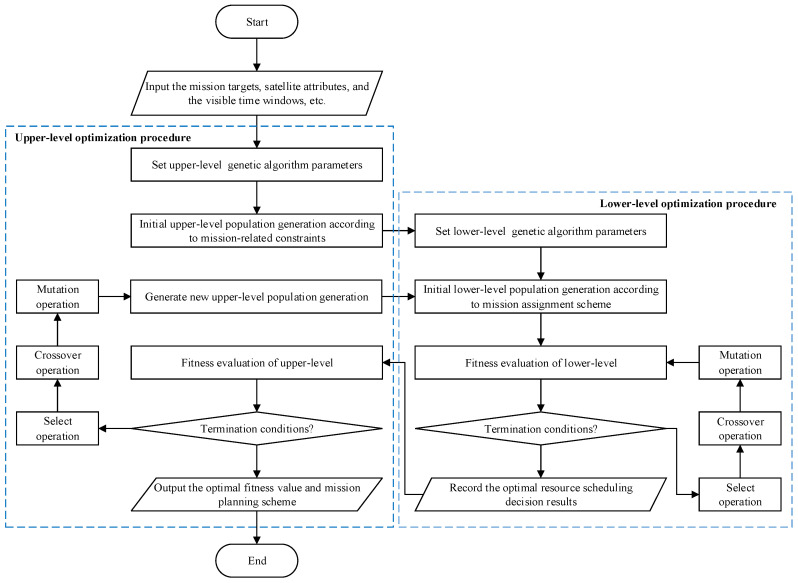
Flowchart of the nested bilevel improved genetic algorithm.

From the above analysis, multi-satellite collaborative mission planning is not only a large-scale complex optimization decision problem but also a complex combinatorial optimization problem. Combined with the constructed bilevel programming model, the obvious NP-hard characteristics make its solution more difficult. The NGA has been proven to be an effective method for solving complex bilevel programming problems [14]. Therefore, a nested bilevel improved genetic algorithm is proposed in this section. After receiving user requirements and preprocessing mission data, mission assignment is first performed by the upper-level optimization. Based on the upper-level results, the lower-level optimization makes a resource scheduling decision, and then the lower level feeds back results to the upper level for further solution. After multiple interactions and iterations, a satisfactory multi-satellite collaborative mission planning scheme is obtained. The implementation process of this algorithm clearly shows the relationship between the upper- and lower-level optimizations, as shown in Figure 2. Throughout the whole decision-making process, the output of the upper level is the input of the lower level, and meanwhile, the output of the lower level is also the input of the upper level. The step-by-step implementation process of the algorithm is outlined as follows:

Step 1: Input. Input related data including observation mission requirement data, satellite data, and others. Parameters such as the population size, the maximum number of iterations, the maximum and minimum values of crossover and mutation probabilities of the upper- and lower-level genetic algorithm are set, and the algorithm begins.

Step 2: Upper-level population initialization. Missions and satellite resources are encoded according to the upper-level encoding strategy, a feasible solution set is constructed based on constraints such as mission types, mission resolutions, and mission visibility, and then combinations from the feasible solution set are randomly selected to generate the initial population for the upper level.

Step 2.1: Lower-level population initialization. The upper-level planning is used as a foundational parameter for the lower-level planning, where an observation time window is randomly assigned to each satellite for the missions to which it is allocated based on the upper-level population. This process includes encoding to generate an initial lower-level population, and auxiliary encoding is incorporated to better handle the constraints of the lower level.

Step 2.2: Lower-level fitness evaluation. The fitness values of the lower-level population are calculated through the fitness function and sorted.

Step 2.3: Lower-level termination condition determination. It is determined whether the maximum number of iterations of the lower level is reached. If so, the individual with the highest fitness value is provided as output, and the process proceeds to Step 3. Otherwise, the process enters the lower-level genetic operation in Step 2.4.

Step 2.4: Lower-level genetic operations. Selection, crossover and mutation operations are performed on the lower-level population. A tournament selection method and an elitism retention strategy are used for selection operations. Two-point crossover with adaptive crossover probabilities and simple mutation with adaptive mutation probabilities are utilized to generate new individuals for the next generation, and then the process continues to iterate in Step 2.2.

Step 3: Upper-level fitness evaluation. Once the lower level reaches its termination condition and the optimal individual is fed back to the upper level, the fitness values of the upper-level population are calculated and sorted.

Step 4: Upper-level termination condition determination. It is determined whether the upper-level termination condition is met. If the upper-level model has undergone multiple generations of selection, crossover, and mutation genetic operations and has largely completed global search and local optimization, and it is difficult to find better individuals, the algorithm can be terminated and can proceed to Step 6. Otherwise, the algorithm enters the upper-level genetic operation in Step 5 to generate a new population for further iteration.

Step 5: Upper-level genetic operations. Firstly, the elite retention strategy is used to directly retain some excellent individuals, and then the population is selected based on the tournament selection method. Two-point crossover is performed based on the adaptive crossover probabilities, and simple mutation is performed based on the adaptive mutation probabilities. Thus, new individuals are generated to form a new upper-level generation. Then, the algorithm enters Step 2.1 to perform lower-level scheduling and begin the optimization of the new population generation.

Step 6: Output. The optimal mission planning scheme and fitness value are outputted, and the algorithm ends.

#### 2.3.1. Chromosome Encoding

**Figure 3:**
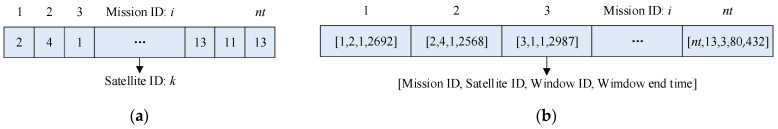
The bilevel chromosome encoding method. (**a**) Chromosome representation of the upper-level optimization; (**b**) chromosome representation of the lower-level optimization.

Chromosome encoding is the key to genetic algorithms, and it expresses the solution of a problem in the form of chromosomes. The upper-level optimization uses natural number encoding method to represent the mission assignment, encoding with the mission number and satellite number information. The gene index is the mission id, the chromosome length is the number of missions, and the gene value represents the satellite id assigned to the mission, as shown in Figure 3a. For better transmission of the upper-level population information and, meanwhile, to conveniently feed back the specific execution time of missions to the upper level, the lower-level optimization’s chromosome encoding also uses the mission id as the gene index, as shown in Figure 3b. Each gene value contains a set of information composed of four parts: the mission id, satellite id, window id, and window end time.

The selection of the initial population largely determines the correctness of the execution results and the running efficiency of the algorithm. Typically, the initial population in genetic algorithms is generated globally and randomly, which ensures that the initial population has a certain diversity. However, in the face of actual large-scale and multi-constraint complex problems, a completely random initial population may lead to a large initial solution space, resulting in low optimization efficiency. Furthermore, it is difficult to achieve the completeness of constraint processing, resulting in significant deviations in the results. Therefore, the heuristic generation method is used to initialize the population in the upper-level model.

Firstly, based on the constraints, such as the type, resolution, and visibility of missions, a feasible solution set of satellites that satisfy the constraints is constructed. Then, a satellite is randomly selected for each mission from the feasible solution set, and several chromosomes satisfying the mission-related constraints are formed according to the encoding rules. Finally, an initial population composed of several individuals is formed. The population initialization based on heuristic rules not only deals with the mission-related constraints well, providing feasible mission assignment schemes as the input of the lower level, but also avoids the algorithm having a large search space, which ensures the running efficiency and convergence speed of the algorithm to a certain extent.

#### 2.3.2. Fitness Function

A fitness function is an indicator used in genetic algorithms to judge the quality of individuals in a population and is the basis of genetic operations. The upper-level optimization primarily aims to maximize the mission profit and mission completion rates while also taking the load balancing of satellite resources into account, and solutions that do not meet the upper-level constraints are excluded during the population initialization. Therefore, the optimization objective of the upper-level model is directly used as the fitness function for the upper-level genetic algorithm. Similarly, the fitness function for the lower-level genetic algorithm is also the optimization objective of the lower-level optimization.

#### 2.3.3. Genetic Operations

Selection, crossover, and mutation are the three basic genetic operations of genetic algorithms, and they jointly promote the evolution of populations, allowing algorithms to explore the solution space and gradually approach the optimal solution. The core idea of genetic algorithms is the optimization of solutions by simulating the process of natural selection, so the genetic operations here are universally applicable to both the upper- and lower-level optimizations.

- Selection operation

This study improves the selection operation of the standard genetic algorithm using a tournament selection method and elite retention strategy. On the one hand, the population diversity can be maintained to avoid the algorithm falling into local optima. On the other hand, superior genes can be transferred to the next generation as much as possible to speed up the convergence of the algorithm.

The tournament selection method simulates the competitive process among individuals in a tournament, continuously selecting winners of the competition to form a new population. This method randomly selects *k* individuals from the current population as contestants, compares the fitness values of the contestants, chooses the individual with the highest fitness value as the winner of that round, and conducts multiple rounds of competition until enough winners are selected. In general, the value of k is 2 or 3.

The elite retention strategy means that the best individuals in the current population are directly retained in the next generation’s population, i.e., a certain number of individuals with the highest fitness values in the current population are copied and directly added to the next generation’s population without any modification by genetic operators in the next evolution. This strategy can effectively ensure that the optimal individuals of the current generation will not be lost due to subsequent genetic operations such as crossover and mutation, and it is conducive to faster convergence of the algorithm.

- Crossover operation

Both the upper and lower levels perform a two-point crossover with adaptive crossover probabilities to promote the genetic diversity of the population and optimize solution exploration. The adaptive ability in genetic algorithms is reflected in a population’s ability to adjust its behavior according to the changes in the surrounding environment. According to the fitness values of the current population, a sine function is used to adaptively improve the crossover probability *P_c_*. Its expression is shown below.

(11) $$P_{c}=\left\{\begin{array}{ll} P_{c}^{max}, & f^{\prime }<f_{avg}\\ P_{c}^{max}-(P_{c}^{max}-P_{c}^{min})\times \sin(\frac{\pi (f^{\prime }-f_{avg})}{2(f_{max}-f_{avg})}), & f^{\prime }\geq f_{avg} \end{array}\right.$$

**Figure 4:**
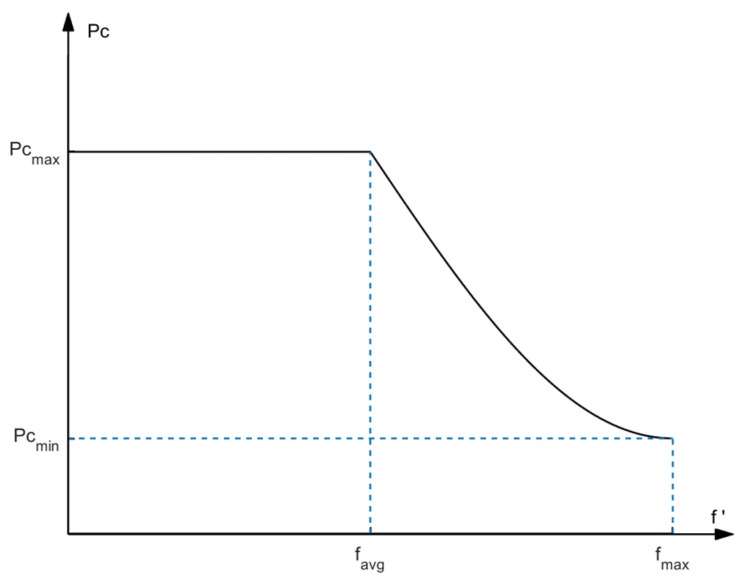
The change curve of the adaptive crossover probability with individual fitness values.

Here, *P_c_^max^* and *P_c_^min^* are the maximum and minimum values of the crossover probability, respectively, *f′* is the maximum fitness value of the two individuals participating in the crossover, *f_max_* is the maximum fitness value of the individuals in the current population, and *f_avg_* is the average fitness value of the current population. With the evolution of the evolutionary process, the curve of the crossover probability after being adaptively adjusted with the change in the individual fitness values is shown in Figure 4.

When the individual fitness is lower than the average fitness of the population, it indicates that the individual is far from the optimal solution, and the global search ability of the algorithm is improved with a higher crossover probability. When the individual fitness value is high, the crossover probability should be reduced to protect good individuals from being destroyed.

**Figure 5:**
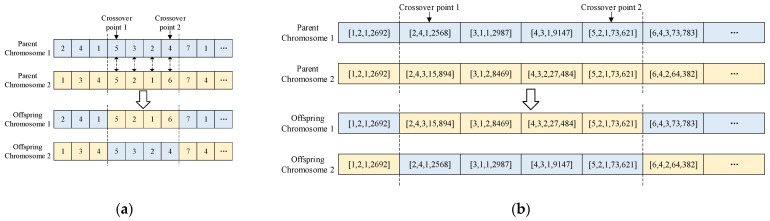
Examples of the two-point crossover operation. (**a**) An example of a crossover operation in the upper-level optimization; (**b**) an example of a crossover operation in the lower-level optimization.

Two-point crossover involves randomly selecting two crossover points on the parent chromosomes, exchanging the gene segments between them according to the crossover probability, and generating offspring individuals with recombined genetic information. An example of the crossover operation for the upper and lower levels is shown intuitively and in detail in Figure 5.

- Mutation operation

**Figure 6:**
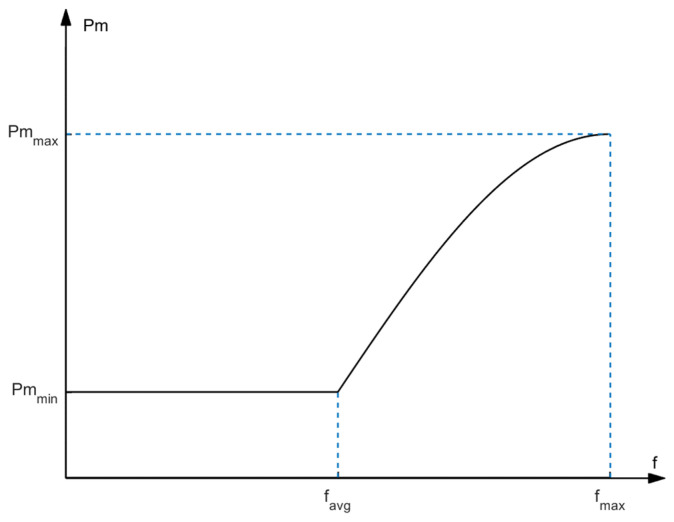
The change curve of the adaptive mutation probability with individual fitness values.

The upper and lower levels perform simple mutation with an adaptive mutation probability to enhance the exploration and development capabilities of the algorithm. According to the fitness values of the current population, similarly, a sine function is used to make adaptive improvements to the mutation probability *P_m_*, and its expression is as follows:

(12) $$P_{m}=\left\{\begin{array}{ll} P_{m}^{min}, & f<f_{avg}\\ P_{m}^{min}+(P_{m}^{max}-P_{m}^{min})\times \sin(\frac{\pi (f-f_{avg})}{2(f_{max}-f_{avg})}), & f\geq f_{avg} \end{array}\right.$$

where *P_m_^max^* and *P_m_^min^* are the maximum and minimum values of the mutation probability, respectively, *f* is the fitness value of the mutated individual, *f_max_* is the maximum fitness value of the individual in the current population, and *f_avg_* is the average fitness value of the current population. With the evolution of the evolutionary process, the curve of mutation probability after being adaptively adjusted with the change in individual fitness values is shown in Figure 6.

When an individual’s fitness is low, a smaller mutation probability is used to avoid excessive randomness in the algorithm. When an individual’s fitness is higher than the average fitness of the population, the mutation probability is increased to maintain the diversity of the population and prevent the algorithm from falling into local optima.

**Figure 7:**
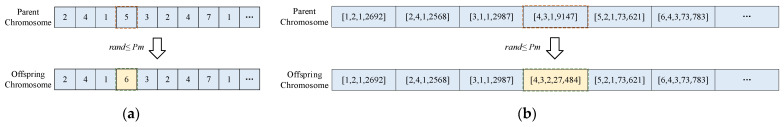
Illustration of simple mutation operations. (**a**) An example of a mutation operation in the upper-level optimization; (**b**) an example of a mutation operation in the lower-level optimization.

Simple mutation operates by randomly selecting a gene bit of an individual and mutating it according to the adaptive mutation probability to produce a new gene, thus forming a new individual. Examples of the upper and lower levels of mutation operations are illustrated in detail in Figure 7.

It is worth noting that during the encoding and population initialization stages, the constraints have been processed, which means that there is a one-to-one correspondence between a point representing an individual in the search space and a point representing a feasible solution in the solution space. Therefore, throughout the entire algorithm process, invalid solutions will not be generated because of genetic operations, ensuring the correctness of the results.

#### 2.3.4. Bilevel Interaction

The interaction between the upper and lower levels reflects the core of the nested bilevel genetic algorithm; the upper-level model, as the leader, is responsible for global decision making and formulating the mission assignment plans. The lower-level model acts as a follower, optimizing locally based on the decisions of the upper level and scheduling satellite resources to arrange the specific execution times of missions. This interaction ensures that the upper level’s global strategy is coordinated and consistent with the lower level’s local execution, continuously optimizing decisions through the iterative process until the optimal or satisfactory solution that meets all constraints is found. This interaction not only improves the efficiency and resource utilization rate of mission scheduling but also enhances the adaptability and robustness of the algorithm for complex multi-satellite collaborative mission planning problems.

## 3. Results

To verify the rationality and effectiveness of the proposed bilevel planning model and the nested bilevel improved genetic algorithm in the multi-satellite collaborative observation mission planning problem, simulation experiments are conducted.

### 3.1. Experimental Environment and Data

This study simulated 10 satellites and 200 mission targets as the basic data for the experiment. The targets are randomly distributed in a geographical range between 60 degrees north and south in latitude around the globe, and the simulation time is 24 h. Information on the relevant attributes of the satellite resources is presented in Table 2.

In the simulation experiment, the population size for both the upper- and lower-level solutions is set to 20, the number of iterations is 50, and the maximum crossover and mutation probabilities are 0.7 and 0.08, respectively, with minimum probabilities of 0.3 and 0.01. The iteration termination conditions for both iterations are set to either reaching the number of iterations or when an optimal value persisted five consecutive times.

### 3.2. Performance Evaluation of NBIGA

**Figure 8:**
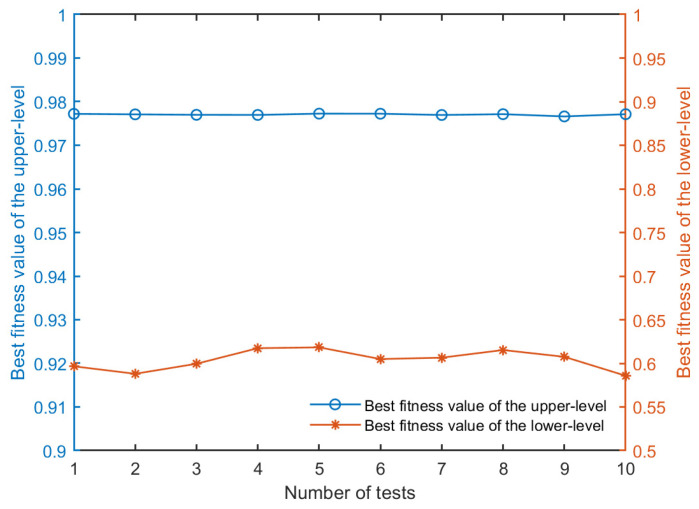
Results of the performance evaluation of the NBIGA.

To test the stability of the proposed NBIGA, 10 independent test experiments are conducted in a simulation scenario with a mission scale of 100. In each experiment, the algorithm is performed under the same initial conditions, and the optimal fitness values of the upper and lower levels are obtained, as shown in Figure 8. The upper-level fitness values, which include mission profit, mission completion, and resource load balance, have average, maximum, and minimum values of 0.9771, 0.9772, and 0.9766, respectively, over these 10 tests. The percentage increase or decrease from the average to the maximum and minimum values are 0.02% and 0.05%, respectively. The lower-level fitness values, which reflect the mission completion time, have averages, maximums, and minimums of 0.6040, 0.6183, and 0.5858, respectively. The percentage increase or decrease from the average to the maximum and minimum values are 2.37% and 3.02%. The results show that the percentage change is not significant, indicating that the algorithm is relatively stable.

### 3.3. Simulation Experiment Analysis

**Figure 9:**
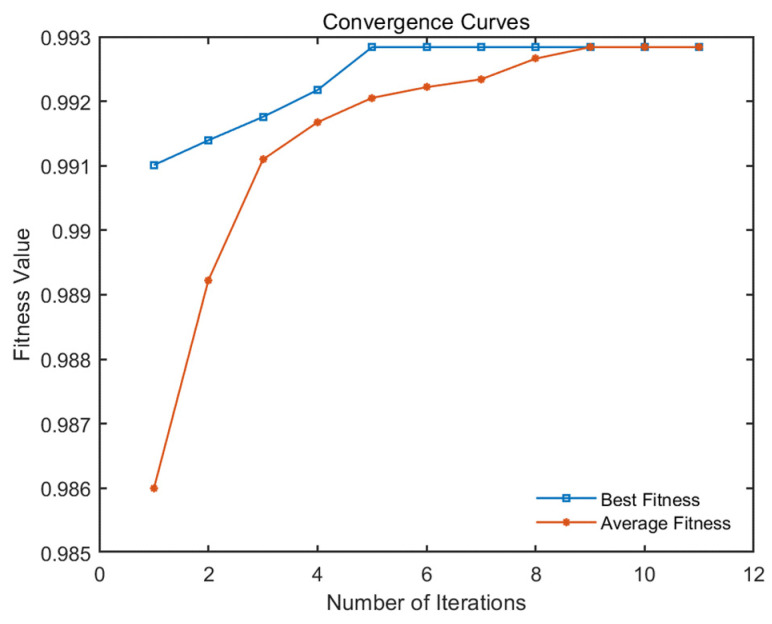
Convergence curves of the fitness value with the number of iterations.

**Figure 10:**
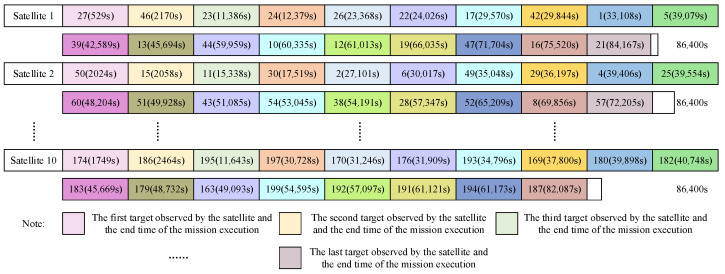
Multi-satellite collaborative mission planning results.

Based on the experimental scenarios and dataset described in Section 3.1, the curves of the changes in fitness values with the number of iterations are shown in Figure 9, which display both the best and average fitness values. According to the termination conditions, the loop exits at the 12th iteration, achieving an optimal fitness value of 0.9928, with 199 tasks being completed in 43.99 s. In addition, Figure 10 intuitively shows the scheduling results of this multi-satellite collaborative mission planning, and the evidence shows that each type of satellite collaboratively performs corresponding missions in a relatively equal number. Taking satellite 1 as an example, the sequence of missions to be observed by satellite 1 is the following: [27, 46, 23, 24, 26, 22, 17, 42, 1, 5, 39, 13, 44, 10, 12, 19, 47, 16, 21]; it completes all assigned tasks at 23 h, 37 min, and 12 s within a one-day planning period. The comprehensive analysis shows that the average fitness value obtained with the improved algorithm is very close to the optimal fitness value, and the running time is within 1 min, indicating that it can respond to the user needs quickly while ensuring better scheduling results. This demonstrates the rationality and effectiveness of the bilevel programming model and the NBIGA established in this study.

To further analyze the performance of the proposed NBIGA, a set of comparative experiments are conducted based on the experimental scenarios and dataset described in Section 3.1, and the algorithm and the nested bilevel basic genetic algorithm (NBGA) are applied. The general framework of the NBGA is the same as that of the algorithm proposed in this study, with the difference being that the genetic algorithm is basic, adopting the roulette wheel selection method and fixed crossover and mutation probabilities, which are 0.6 and 0.05, respectively. In addition, the nested bilevel basic genetic algorithm does not use a random way to initialize the population—but is the same as the NBGA—to ensure that a better solution can be found in a more effective time. The results obtained after running both algorithms 10 times each are shown in Table 3. The results show that the total revenue of the NBIGA is 2.11% higher than that of the NBGA, the fitness value of the lower-level resource scheduling is 1.24% higher, and the running time of the algorithm is reduced by 65.83%. To summarize, the NBIGA could not only obtain more stable and better solutions but also reduce the time required, responding quickly to user needs.

**Figure 11:**
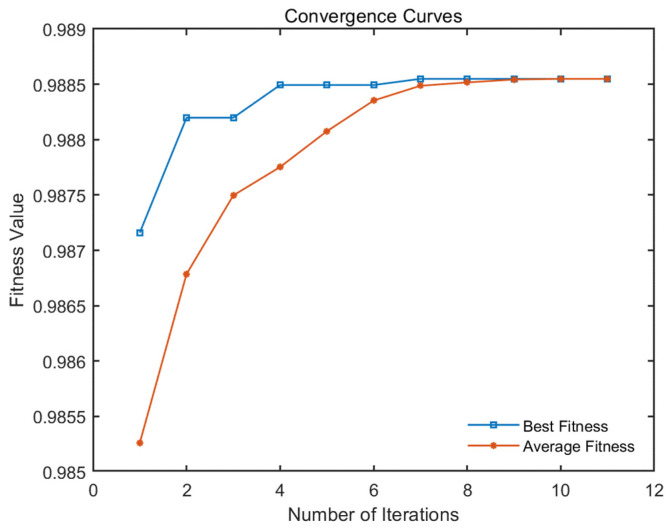
Convergence curves of the fitness value with the number of iterations for 400 missions.

Furthermore, we expand the mission scale to 400 for simulation experiments and obtain the curves of the fitness value as they change with the number of iterations, as shown in Figure 11, which shows the optimal and the average fitness values. The experimental results show that the optimal fitness value of the algorithm is 98.86%, with 397 tasks being completed in 90.26 s. The results show that with the increase in the mission scale, the algorithm could still quickly find the optimal value and meet the termination conditions to exit the loop with a high mission completion rate. Moreover, for each type of satellite, reasonable mission assignment makes the load of the same type of satellite relatively balanced. The rationality and effectiveness of the bilevel programming model and the NBIGA proposed in this study are further verified for the multi-satellite collaborative mission scheduling problem.

## 4. Discussion

With the sharp increase in users’ demand for Earth observation and the number of in-orbit satellites, solving the problem of multi-satellite collaborative mission planning has become the key to meet users’ needs and fully utilizing in-orbit satellite resources. To address the issue, we propose a bilevel programming model and the NBIGA for the solution. The bilevel programming model for multi-satellite collaborative mission planning considers the internal interactions between the two decision-making stages of mission assignment and resource scheduling. This model recognizes that these two stages are not isolated but are, in fact, deeply interconnected, with decisions in one stage significantly influencing the outcomes in the other. For this reason, we adopt and improve a NGA which can naturally accommodate this structure by employing one genetic algorithm for the upper-level decisions (mission assignment) and another for the lower-level decisions (resource scheduling), and propose the NBIGA to better suit for the solution of the multi-satellite collaborative mission planning problem.

Compared with the NBGA, the algorithm proposed in this study fully considers the shortcomings of genetic algorithms and the features of the problem we are addressing, aiming at enhancing solution quality, computational efficiency, and adaptability to the complex nature of multi-satellite collaborative mission planning. Specifically, the NBIGA employs adaptive mechanisms to adjust crossover and mutation probabilities based on the population’s fitness. This adaptive approach enables the algorithm to better explore the solution space during the initial phases and fine-tune solutions as it converges. And by incorporating an elitism strategy, the NBIGA ensures that the best individuals are carried over to the next generation, preserving the best solutions found so far and accelerating convergence. Moreover, more sophisticated and suitable encoding schemes are used in the NBIGA and the initial population are optimized based on heuristic regulation, allowing for a more efficient representation of the solution space and reducing the likelihood of generating infeasible solutions.

From the results of performance evaluation of the NBIGA, the fitness values of the global optimization objective in the test scenarios have very subtle variation no more than 0.1%, which indicates that the NBIGA can provide reliable and consistent results for solving the model and the algorithm’s robustness and its suitability for the intricate planning needs of multi-satellite systems have been validated.

From the simulation results, the superiority of the NBIGA in terms of solution quality and computational efficiency are demonstrated. The NBIGA can achieve an average improvement of 2.11% in solution quality and reduce computation time by 65.83%, compared to the NBGA. And the results show that the NBIGA can not only ensure a high mission completion degree and mission profit rate but also balance the load of satellite resources as much as possible and keep the utilization rate of satellite resources relatively high. Furthermore, the experiment has successfully shown that the NBIGA maintains its effectiveness and efficiency even as the complexity and scale of the mission planning problem increases. This indicates that the algorithm is scalable and can handle larger and more intricate mission planning missions, which is crucial for real-world applications involving multiple satellites.

Although the bilevel programming model and the NBIGA proposed in this study can obtain good results, there is still potential for further improvement. In practical applications, it is difficult to simply deal with user requirements, such as regional target coverage and moving target tracking with point targets, and these kinds of user requirements necessitate an efficient multi-satellite collaborative mode. Therefore, the consideration of multi-satellite collaborative mission planning for regional targets or moving targets will be the focus of our next research.

## 5. Conclusions

Numerous studies have been performed to address the intricate challenges in view of the multi-satellite collaborative mission planning problem. Building upon these foundational efforts, our work introduces a novel perspective by applying bilevel programming to this complex problem. This study investigates the optimization of mission assignment and resource scheduling in multi-satellite collaborative mission planning while considering the inherent interaction between the two decision-making stages. The mission assignment stage establishes the global optimization objectives and decides the mission assignment schemes, while the resource scheduling stage decides the satellite scheduling schemes under the guidance of the global optimization objectives. The two stages are inseparable and complementary. In this study, based on game theory, the problem is transformed into a bilevel programming problem. The upper level corresponds to the mission assignment stage, aiming at maximizing the mission profit rate, the mission completion rate, and resource load balance as optimization objectives, and it is responsible for formulating the mission assignment plans and passing them to the lower level. The lower level corresponds to the resource scheduling stage. Under the premise of obeying the decision of the upper level, a resource scheduling plan is formulated to maximize the resource utilization rate as the optimization goal, and then the scheduling results are fed back to the upper level to assist in decision-making optimization. Subsequently, the NBIGA is proposed, which designs suitable encoding schemes, formulates population initialization rules, and improves genetic operations in detail. This algorithm, having been verified stable and scalable, can effectively solve the bilevel programming model established in this study, as it can realize the comprehensive optimization of mission- and resource-related benefits. Further, the whole scheduling and planning process of the algorithm takes less time, so a rapid response to user needs can be realized as well. This study provides technical support from the perspective of game theory for research on multi-satellite collaborative mission planning methods.

## Figures and Tables

**Table 1:** Notations and definitions.

Notation	Definition
*i*	An index ranging from 1 to *m* represents the mission id, and *m* represents the total number of missions.
*j*	An index ranging from 1 to *n* represents the satellite id, and *n* represents the total number of satellites.
*T*	*T* = {*t*_1_, *t*_2_, …, *t_m_*}, where *T* represents the set of missions.
*S*	*S* = {*s*_1_, *s*_2_, …, *s_n_*}, where *S* represents the set of satellites.
*P*	*P* = {*p*_1_, *p*_2_, …, *p_m_*}, where *P* represents the set of mission profits.
*Res*	*Res* = {*c_r,_*_1_, *c_r,_*_2_, …, *c_r,m_*}, where *Res* represents the resolution set of missions.
*Dur*	*Dur* = {*c_d,_*_1_, *c_d,_*_2_, …, *c_d,m_*}, where *Dur* represents the observation duration set of missions.
*Req*	*Req* = {*c_q,_*_1_, *c_q,_*_2_, …, *c_q,m_*}, where *Req* represents the imaging type requirement set of missions, and Req ∈ {A, B, C, D}, representing the visible light, infrared, hyperspectral, and synthetic aperture radar (SAR), respectively.
*c_l,j_*	*c_l,j_* represents the payload carried by the *j*-th satellite, and *c_l,j_* ∈ {A, B, C, D}, representing the visible light, infrared, hyperspectral, and SAR, respectively.
*c_s,j_*	*c_s,j_* represents the minimum resolution of the *j*-th satellite.
*c_p,j_*	*c_p,j_* represents the available power-on time of the *j*-th satellite.
*c_a,j_*	*c_a,j_* represents the attitude adjust time of the *j*-th satellite.
*v_ijh_*	*v_ijh_* = {*v_s,ijh_*, *v_e,ijh_*}, where *v_ijh_* represents the *h*-th visible time window that the *j*-th satellite can observe the *i*-th mission target, *h* represents the number of the window, which ranges from 1 to *k*, *v_s,ijh_* represents the start time of the *h*-th visible time window, and *v_e,ijh_* represents the end time of the *h*-th visible time window.
*r*	*r* = {*r*_1_, *r*_2_, …, *r_m_*}, where *r* represents the set of satellites assigned to each mission, reflecting the mission assignment scheme.
*o*	*o* = {*o*_1_, *o*_2_, …, *o_m_*}, where *o* represents the set of actual start times when the mission is executed, reflecting the resource scheduling plan.
*x_ij_*	*x_ij_* is a binary variable, representing the results of mission assignment, taking a value of 1 if the *i*-th mission is assigned to *j*-th satellite and 0 otherwise.
*y_ij_*	*y_ij_* is a binary variable, represents the results of resource scheduling, taking a value of 1 if the *j*-th satellite successfully observes the *i*-th mission target and 0 otherwise.

**Table 2:** Information on satellite resource attributes.

Satellite ID	Load Type	Semimajor Axis *a* (km)	Eccentricity *e* (°)	Inclination *i* (°)	Right Ascension of the Ascending Node Ω (°)	Argument of Perigee ω (°)	True Anomaly φ (°)	Resolution (m)
1	Visible light	7171.393	0	96.576	175.72	0	0.075	3
2	Visible light	7171.393	0	96.576	115.72	0	60.075	1
3	Visible light	7171.393	0	96.576	55.72	0	120.075	0.5
4	Hyperspectral	7171.393	0	96.576	145.72	0	30.075	0.5
5	Hyperspectral	7171.393	0	96.576	85.72	0	90.075	0.3
6	Hyperspectral	7171.393	0	96.576	25.72	0	150.075	1
7	Infrared	7083.140	0	98.213	210	0	72	0.5
8	Infrared	7241.140	0	98.877	144	0	200.035	1
9	SAR	7023.140	0	97.971	230	0	324	2
10	SAR	7034.140	0	98.015	115	0	36	0.8

**Table 3:** The results of the comparison of the algorithms.

Algorithms	The Average of the Upper Optimal Fitness Values	The Average of the Lower Optimal Fitness Values	The Average Runtime
NBIGA	99.24%	56.43%	41 s
NBGA	97.13%	55.19%	120 s

## Data Availability

The data presented in this study are all contained within this article.
